# Respiratory microbiota maturation enables machine learning based age prediction in chickens

**DOI:** 10.1186/s12866-026-04947-3

**Published:** 2026-03-18

**Authors:** Alejandro Moreno-León, Ane López-Morales, Ursula Höfle, Marta Barral, Natàlia Majó, José Luis Lavín

**Affiliations:** 1 Centre de Recerca en Sanitat Animal (CReSA), Campus de la Universitat Autònoma de Barcelona (UAB), Unitat Mixta d’Investigació IRTA-UAB en Sanitat Animal, Bellaterra, Catalonia 08193 Spain; 2 Programa de Sanitat Animal, Centre de Recerca en Sanitat Animal (CReSA), IRTA, Campus de la Universitat Autònoma de Barcelona (UAB), Bellaterra, Catalonia 08193 Spain; 3 Applied Mathematics Department, NEIKER, Basque Institute for Agricultural Research and Development, Basque Research and Technology Alliance (BRTA), Bizkaia Science and Technology Park 812L, Derio, Bizkaia 48160 Spain; 4 Animal Health Department, NEIKER, Basque Institute for Agricultural Research and Development, Basque Research and Technology Alliance (BRTA), Bizkaia Science and Technology Park 812L, Derio, Bizkaia 48160 Spain; 5 Grupo Sanidad y Biotecnología (SABIO), Instituto de Investigación en Recursos Cinegéticos IREC (CSIC-UCLM-JCCM), Ciudad Real, Spain; 6 Departament de Sanitat i Anatomia Animals, Facultat de Veterinària Universitat Autònoma de Barcelona (UAB), Campus de la UAB, Bellaterra, Catalonia 08193 Spain

**Keywords:** Poultry, Respiratory Microbiota, Age, Random Forest, Machine Learning, Biomarkers

## Abstract

**Background:**

Understanding how the respiratory microbiota matures with age is key to improving poultry health and pathogen surveillance, yet the ecological processes shaping this transition remain elusive. We aimed to develop an interpretable machine-learning framework capable of identifying age-associated microbial signatures within the chicken nasal microbiota across heterogeneous datasets.

**Results:**

We compiled data from five independent chicken studies and normalized microbial abundances using Counts Per Million (CPM). To address dataset imbalance and ensure cross-study generalizability, we implemented SMOTE over-sampling and a Leave-One-Study-Out (LOSO) cross-validation framework. Within this architecture, we utilized Recursive Feature Elimination (RFE) to identify a stable consensus signature composed of taxa persisting in at least 70% of the iterations. We benchmarked five algorithms: Classification and Regression Trees (CART), k-nearest neighbors (kNN), Support Vector Machines (SVM), Random Forest (RF), and Extreme Gradient Boosting (XGBoost). RF emerged as the best model, achieving a balanced accuracy of 0.965 and a Kappa of 0.920. Consequently, the contribution of each feature was quantified through SHapley Additive exPlanations (SHAP) values on the selected RF model, enabling transparent interpretation of age-dependent microbial patterns. This approach distilled a compact set of predictive taxa, including *Corynebacterium*, *Kocuria*, and members of the *Micrococcaceae*. External validation with longitudinal samples from a Highly Pathogenic Avian Influenza Virus (HPAIV) infection confirmed full generalization, with all 57 samples from 22 chickens correctly classified even under viral-induced conditions.

**Conclusions:**

The proposed workflow combining LOSO-based feature selection, class-balancing, and interpretable machine learning provides a transferable framework for microbiota-based age inference. Such approaches may inform health monitoring, management of poultry production systems, and wildlife surveillance, illustrating the power of interpretable artificial intelligence to reveal conserved host-microbe dynamics across avian systems.

**Graphical Abstract:**

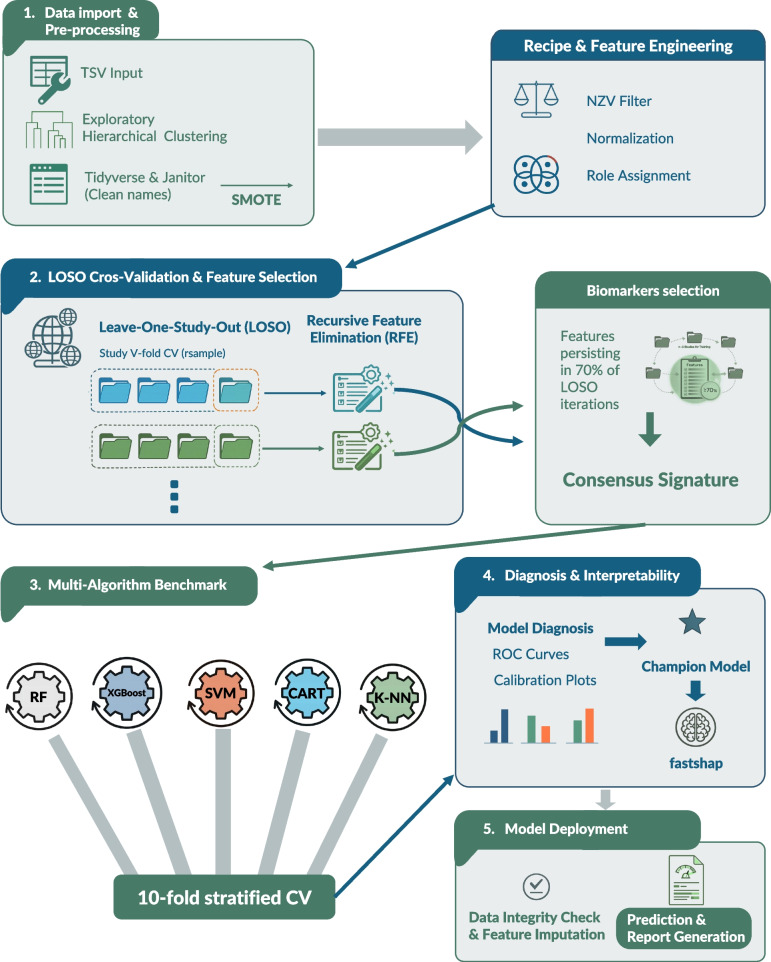

**Supplementary Information:**

The online version contains supplementary material available at 10.1186/s12866-026-04947-3.

## Introduction

The poultry respiratory microbiota varies markedly with bird age, anatomical site, and production stage, shaping community quantity and composition from the upper respiratory tract to the lungs [1–3]. Environmental and management conditions, including farm ammonia levels and, particulate matter concentration and litter practices consistently reshape respiratory bacterial communities and inflammatory response, particularly in the lungs [4–9].

Among the different regions of the avian respiratory tract, the nasal cavity represents a primary site of microbial colonization and early host–microbe interactions. Understanding its age-related dynamics is therefore essential for contextualizing respiratory health and disease susceptibility in poultry. Despite growing interest in the poultry respiratory microbiota, age-associated patterns remain poorly defined. Previous studies have described shifts in nasal *Lactobacillus* abundance [1, 2], but their single-breed design limits broader generalization. Moreover, cross-study heterogeneity and methodological variation have largely restricted comparative analyses to broad, phylum-level trends. Addressing these challenges requires analytical approaches capable of integrating heterogeneous data and revealing subtle, biologically relevant patterns.

Machine learning (ML) has become a powerful approach for extracting meaningful patterns from complex microbiological datasets, which often involve high-dimensional measurements such as genomic, proteomic, or metabolomic profiles. Unsupervised learning methods, such as Principal Component Analysis (PCA) and hierarchical clustering, are frequently applied to reveal intrinsic data structure without predefined labels. PCA reduces dimensionality by projecting samples into a space that captures the greatest variance, facilitating visualization of microbial community differences or strain-level variability [10]. Similarly, hierarchical clustering groups samples based on similarity, enabling the identification of natural clusters such as microbial taxa or phenotypic subgroups [11].

Supervised ML algorithms extend these capabilities by constructing predictive models from labeled data. Decision tree methods like Classification and Regression Trees (CART) provide interpretable classification rules based on microbial features [12]. Instance-based learning, such as k-nearest neighbors (kNN), classifies samples according to proximity in feature space, making it suitable for phenotype prediction [13]. More advanced models, including support vector machines (SVMs) [14], random forests (RF) [15], and gradient boosting algorithms such as Extreme Gradient Boosting (XGBoost) [16], offer robust classification and feature selection capabilities, even in noisy or imbalanced microbiological datasets. Collectively, these approaches allow researchers to uncover hidden biological patterns and to build predictive frameworks for diagnostics, microbial ecology, and antimicrobial resistance studies.

Recently, the growing volume of chicken microbiome studies has enabled age classification in our last work [3] however, cross-study heterogeneity and data complexity have largely limited insights to broad, phylum-level trends. To address this, we apply a novel machine-learning approach to extract age-associated patterns at genus-level resolution across heterogeneous datasets. Using this framework, we also trained and externally validated predictive model that infers host age from the chicken nasal microbiota.

## Materials and methods

### Data acquisition

Raw data were compiled as described in our previous work [3]. Data processing and taxonomic assignment followed the same pipeline detailed in that manuscript. Briefly, primer trimming was performed with cutadapt [17], and denoising was conducted in QIIME2 (v2024.10) [18] using qiime dada2 denoise [19]. Feature tables and representative sequences were merged using qiime feature-table merge and qiime feature-table merge-seqs [18]. Taxonomic classification was carried out with the SILVA 138 database [20] using the qiime feature-classifier classify-sklearn command [21]. Samples whose rarefaction curves (Shannon index) did not reach the plateau were excluded. For downstream analyses, ASVs were collapsed to genus level (taxonomic rank 6) using qiime taxa collapse [18], generating the final feature table used to train the machine-learning models.

### Exploratory hierarchical clustering analysis

Between-sample dissimilarities were then computed using the Bray–Curtis index [22] for exploratory hierarchical clustering analysis. Prior to distance calculation, count data were normalized using counts per million (CPM). To ensure compatibility of this non-metric dissimilarity with Ward’s minimum-variance (Ward.D2) method, we applied a square-root transformation to the Bray–Curtis matrix, yielding a metric form appropriate for Ward’s criterion Hierarchical clustering (Ward.D2) [23, 24] was then performed on the transformed matrix and visualized as a dendrogram with samples labeled by group.

### Initial processing and feature engineering

Our analysis began by importing raw taxonomic abundance tables through the *tidyverse* framework [25]. We addressed nomenclature inconsistencies by applying the *clean_names()* function from the janitor package, which ensured a uniform syntactic structure across all features. Before downstream modeling, abundances were transformed to a numeric scale to maintain mathematical stability. We then built a comprehensive preprocessing “recipe” using the *recipes* package. This workflow relied on three specific logic gates: first, we applied Zero Variance (ZV) filtering via *step_nzv()* to discard non-informative variables; second, we used *step_normalize()* to center and scale numeric predictors; and third, we prevented data leakage by designating sample and study identifiers as metadata through *update_role().* Both outcomes and study IDs were defined as categorical factors. To counter the inherent bias of unbalanced datasets, we utilized the Synthetic Minority Over-sampling Technique (SMOTE) [26] via the *themis* library [27].

### Validation strategy and biomarker discovery

We evaluated the generalizability of microbial signatures using a Leave-One-Study-Out (LOSO) cross-validation framework [28, 29]. By employing *group_vfold_cv*() from the *rsample* package [30], we iteratively reserved one cohort for external testing while training on the remaining *n-1* studies. Within this iterative LOSO architecture, we performed Recursive Feature Elimination (RFE) using the *ranger* engine [31]. This process allowed us to isolate and rank the most informative taxa based on Gini impurity. To establish a stable “consensus” signature, we retained only the biomarkers that persisted in at least 70% of the independent LOSO iterations.

### Algorithmic benchmarking and selection

A systematic comparison of five machine learning architectures was executed using the *workflowsets* package [32]. Our ensemble included Random Forest (RF) utilizing 1,000 trees [15], Extreme Gradient Boosting (XGBoost) [16], Support Vector Machines (SVM) [14] with polynomial kernels, Classification and Regression Trees (CART) [12], and k-Nearest Neighbors (kNN) [13]. We optimized these models through 10-fold stratified cross-validation. Model performance was quantified via the *yardstick* package [33], utilizing a metric suite composed of *Accuracy*, *F1-Score*, *Kappa*, *Balanced Accuracy* and *Area Under the Curve* (*AUC*). The architecture demonstrating the highest *Balanced Accuracy* was ultimately designated as our best model.

### Calibration, diagnostics, and interpretability

We went beyond standard accuracy metrics by scrutinizing classification reliability with ROC curves and Windowed Calibration Curves via the *probably* package [34]. This ensured that predicted probabilities were representative of actual clinical risks across the entire spectrum. To cross-verify structural feature importance, we utilized the *vip* package [35]. Furthermore, we resolved the “black-box” nature of the champion model by calculating Shapley Additive Explanations (SHAP) [36]. Using the *fastshap* library [37], we conducted 50 Monte Carlo simulations to determine both the absolute magnitude and the directional influence of each consensus taxon on the final clinical prediction.

### Model deployment and data integrity protocols

We authored a dedicated deployment script to extend the model’s utility to external datasets. This pipeline begins with an automated integrity audit, aligning the taxonomy of new samples with the established model space via *extract_preprocessor()* [32]. To manage the technical heterogeneity across sequencing platforms, we implemented a feature-imputation strategy: taxa absent in new samples are assigned a value of zero to maintain matrix consistency [38]. The system calculates a data integrity score to quantify the biological overlap between training and testing sets. Predictions and confidence metrics are then generated through the *tidymodels* framework, resulting in a visual report that ensures results are only interpreted when they meet specific quality thresholds [34].

### External dataset for model verification

To evaluate our model, we used a dataset obtained from an experimental infection conducted under biosafety level 3 (BSL-3) conditions at IRTA-CReSA (Barcelona, Spain). In this trial, fertilised eggs from commercial Ross 308 broiler chickens were obtained from a collaborating hatchery in Catalonia (Spain). Eggs were incubated and hatched under standard BSL-3 conditions at the IRTA-CReSA high-containment facilities. Upon hatching, chicks were transferred to two experimental boxes for control and infection conditions.

Animals were reared under these controlled conditions until 15 days of age, when they were intranasally inoculated with a highly pathogenic avian influenza virus (HPAIV, subtype H7N1) or mock-inoculated as controls. Choanal swabs were collected from both infected and control birds at 0, 2, 4, and 10 days post-infection (dpi), yielding 57 samples from 22 chickens. At the scheduled endpoints, animals were humanely euthanised using consisting of a intravenous administration of sodium pentobarbital (140 mg/kg body weight). This dataset is publicly available on Zenodo (10.5281/zenodo.17466208).

### Supporting online resources

Table S1 includes a summary of the publicly available online resources used to perform all analyses in this study.

## Results

The dataset used for model training in this study derives from our previous work [3], which originally compiled data from eight independent studies. In the present analysis, we restricted the dataset to five studies with complete and reliable age metadata, as three of the originally screened studies did not report any age information and were therefore excluded. We further focused exclusively on nasal cavity samples. To minimize confounding effects and ensure consistency across studies, only healthy, untreated chickens were included.

### Hierarchical clustering for data quality control and sample stratification

**Fig. 1:**
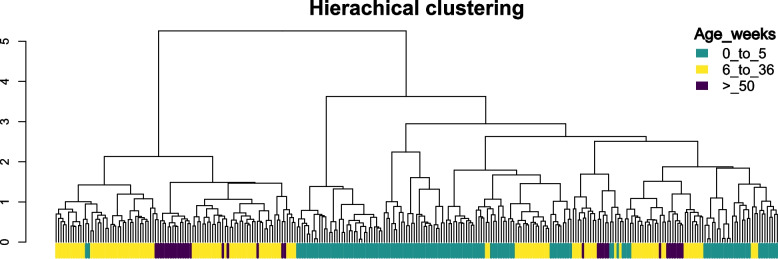
Hierarchical clustering of nasal microbiota samples by age groups based on Bray–Curtis dissimilarity

We applied hierarchical clustering as an exploratory analysis to qualitatively inspect the overall structure of the data. As expected, the unsupervised clustering did not clearly separate samples into three age-defined clusters (Fig. 1), as cluster formation was largely driven by confounding factors such as SPF status and, to a similar extent, host breed. These age categories were originally defined based on alpha-diversity and compositional shifts described in our previous work. Age was also evaluated by PERMANOVA along with other factors, where SPF status and breed demonstrated the highest F-values [3]; however, the strong heterogeneity across studies likely masked age-driven patterns in the unsupervised analysis.

### Study-aware feature selection using LOSO and consensus core definition

Given the strong inter-study heterogeneity observed in exploratory analyses, we adopted a study-aware machine learning framework based on Leave-One-Study-Out (LOSO) cross-validation to assess generalizability. Within each LOSO iteration, RF–based Recursive Feature Elimination (RFE) was applied exclusively to the training data to identify the most informative taxa for age classification.

**Fig. 2:**
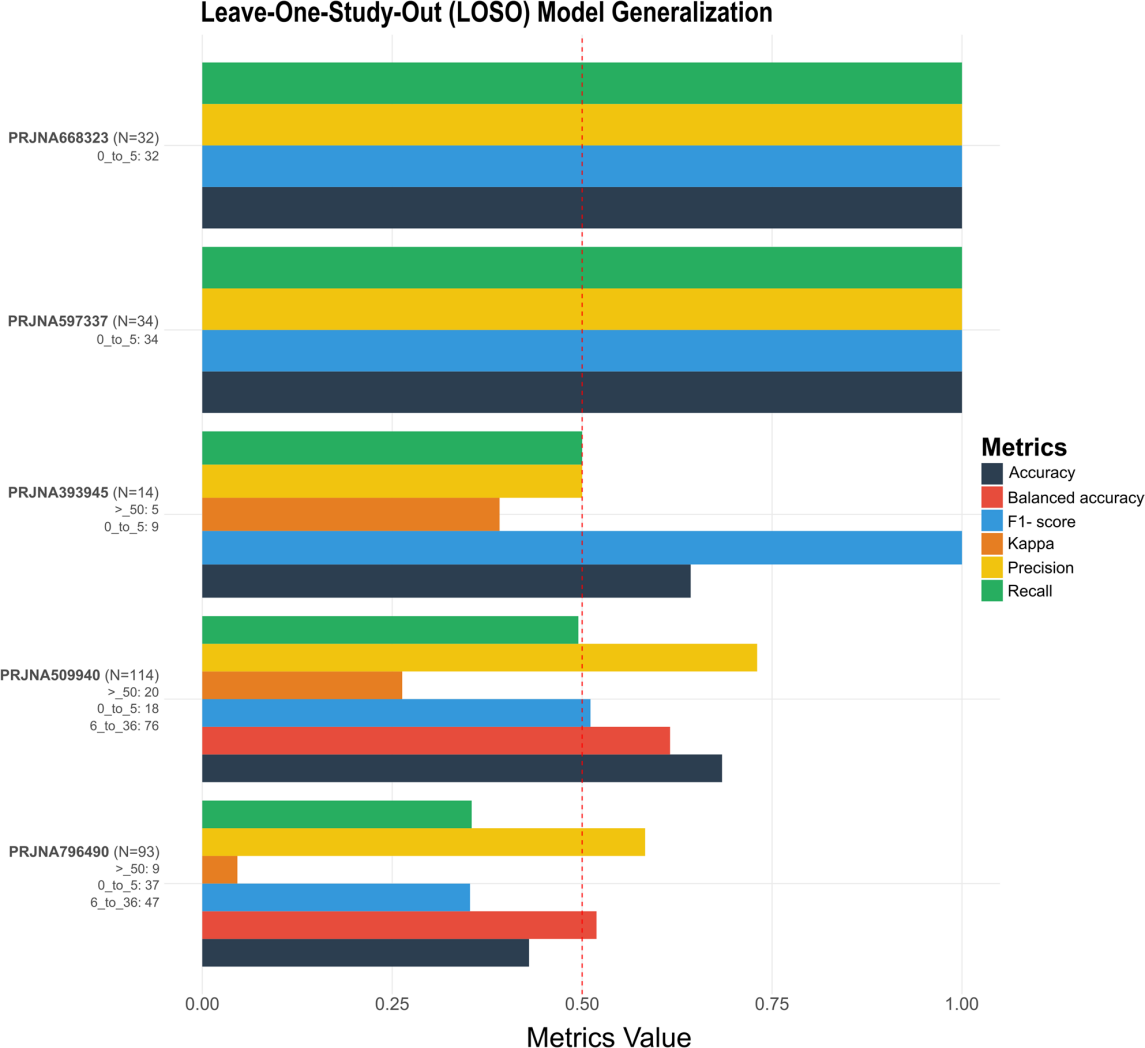
Cross-study generalizability via LOSO validation. The bar plot illustrates the performance metrics (Accuracy, Balanced Accuracy, F1-Score, and Kappa) for each independent study when treated as an external test set. The dashed line indicates the random chance baseline (0.5). The number of samples per age class included in each study is shown below the corresponding study accession number

LOSO evaluation showed that when studies encompassing multiple age categories were held out, model performance decreased noticeably, indicating reduced generalization capacity in these splits (Fig. 2). This effect is partly attributable to the limited number of studies covering a broad spectrum of age classes, which constrains the availability of representative age-related signals when such studies are excluded during LOSO evaluation.

To isolate robust age-associated microbial signatures and mitigate study-specific effects, we defined a Consensus Core composed of taxa selected in at least 70% of independent LOSO iterations, resulting in 19 Features (Table S2). This approach prioritizes cross-study reproducibility over within-study optimization and ensures that downstream modeling relies on features consistently informative across diverse experimental contexts.

### Model selection and benchmarking over the consensus core features

Supervised classification models were trained using the Consensus Core features and benchmarked across five learning algorithms: RF, XGBoost, CART, kNN, and SVM. Model performance was evaluated using stratified cross-validation, with Mean Balanced Accuracy and Cohen’s Kappa used as primary metrics to account for class imbalance.

**Fig. 3:**
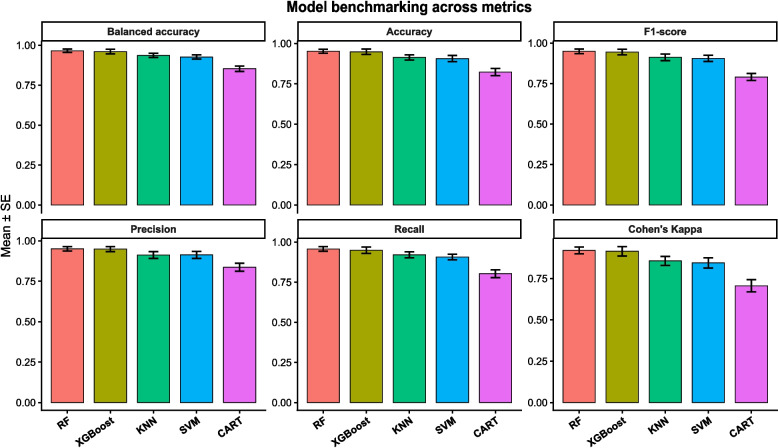
Global consensus benchmark. Comparative performance of five machine learning architectures (RF, XGBoost, SVM, k-NN, and CART). Data represent the mean ± standard error from 10-fold stratified cross-validation on the universal microbial signature

Among the evaluated approaches, RF achieved the best overall performance (Fig. 3 and Table S3) and demonstrated higher probability calibration (Figure S1) and was therefore selected for subsequent analyses.

### Feature biological relevance assessed by SHAP values

**Fig. 4:**
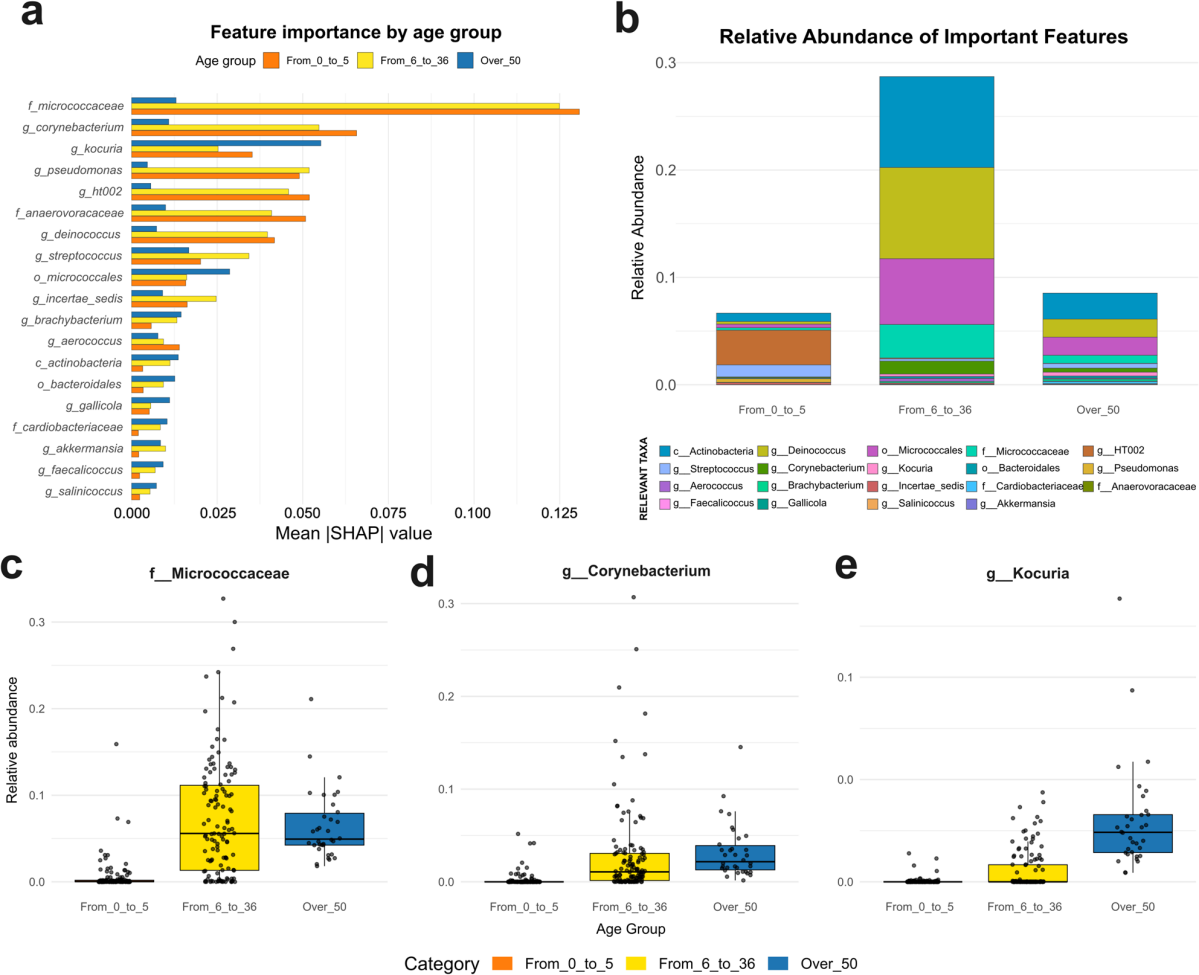
Identification of age-associated microbial signatures through interpretable machine learning. **a** Class-wise feature importance assessed by the Mean Absolute SHAP Value. **b** Mean relative abundance of the top predictive taxa across the three age categories. **c** Boxplot showing the distribution of *Micrococcaceae* relative abundance across age groups. **d** Boxplot showing the distribution of *Corynebacterium* relative abundance across age groups. **e** Boxplot showing the distribution of *Kokuria* relative abundance across age groups

The global contribution of individual features was assessed by calculating the Mean Absolute SHAP Value (MASHV). This was achieved by taking the average of the absolute SHAP scores for each feature, segregated by the corresponding target class. As we can see, the class-wise MASHV barplot highlights a compact set of recurrent taxa driving predictions across groups. Among the most influential taxa, Micrococcaceae, *Corynebacterium*, and *Kokuria* stand out for their high predictive power, reflecting marked differences in their mean relative abundance between groups (Fig. 4a). Altogether, these taxa constitute a distinctive microbial signature that enables accurate classification across the three categories (Fig. 4b). All three top predictive taxa display a similar pattern: they are nearly absent in birds aged 0–5 weeks and become abundant in the remaining two age groups (Fig. 4c-e). Despite this shared trend, *Kocuria* shows the strongest predictive contribution and peaks in abundance in birds older than 50 weeks, suggesting a specific association with late-age microbiota maturation (Fig. 4a and e).

### Cross-dataset verification of the selected model

**Fig. 5:**
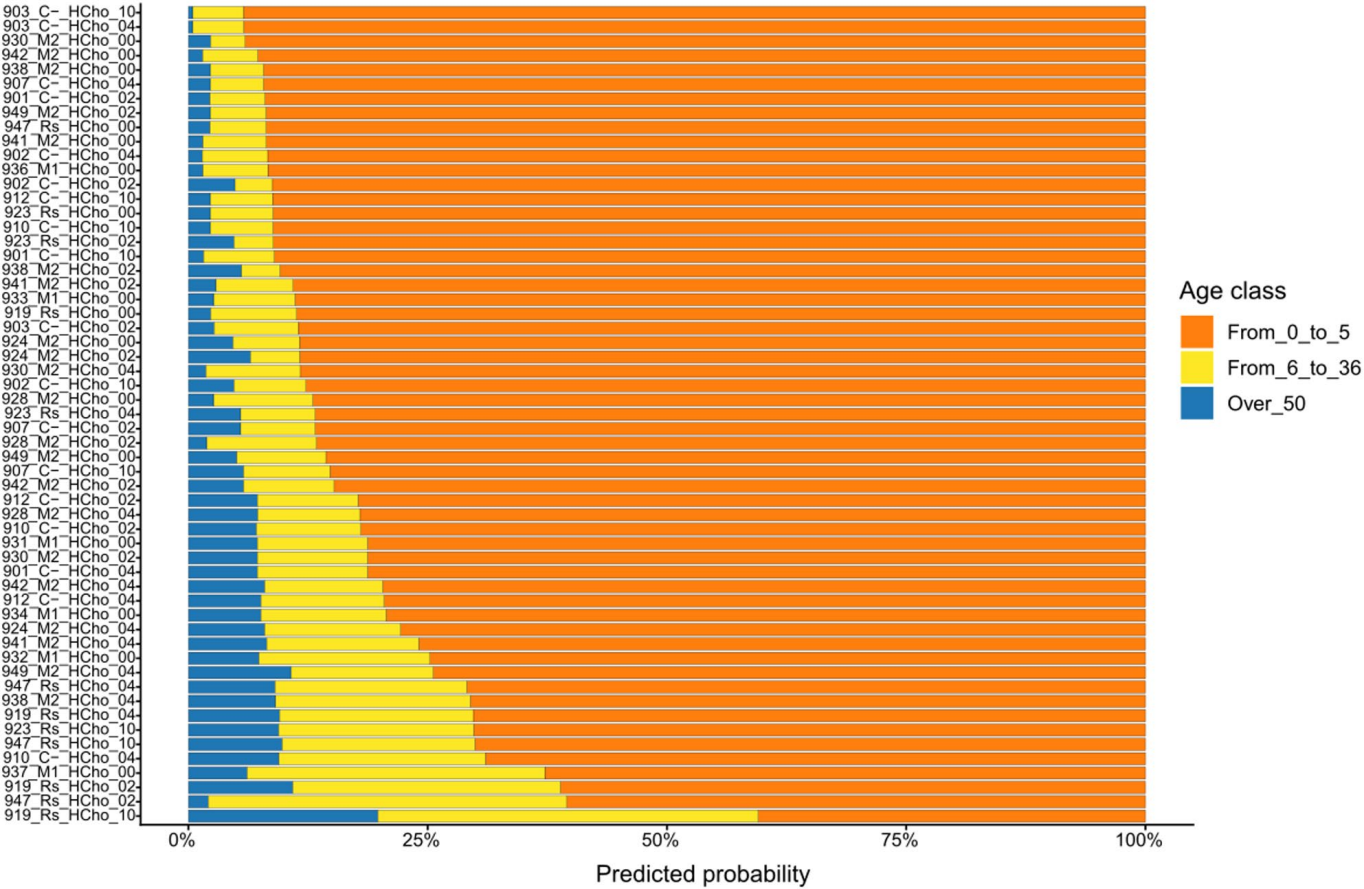
Per-sample predicted class probability distributions in the external validation dataset. Horizontal stacked bar plots display the predicted probabilities generated by the selected RF model for each sample across the three age classes. All samples correspond to birds aged 0–5 weeks, enabling evaluation of model confidence and probability calibration in an external dataset

To test our choice model, we used a dataset obtained from an experimental infection with HPAIV conducted under BSL-3 conditions. All samples in this external dataset corresponded to birds aged from 0 to 5 weeks and were consistently assigned the highest predicted probability to the correct age class (Fig. 5). Importantly, although the dataset comprises 57 observations, these represent longitudinal repeated measures from 22 individual chickens and are therefore not statistically independent at the sample level. This within-individual dependence may inflate apparent performance estimates and should be considered when interpreting the external validation results. Overall, these results support the model’s ability to discriminate the early-life age class under altered microbial conditions associated with viral infection; however, independent validation of the intermediate and adult age categories remains necessary to confirm broader generalization.

## Discussion

In this study, we developed and validated a machine‑learning framework to identify age‑associated microbial patterns in the chicken nasal cavity across heterogeneous datasets. Our results show that, despite substantial variation among studies, breeds, and rearing conditions, the nasal microbiota harbors reproducible, age‑dependent signatures. Although the complexity of microbial interactions makes it impossible to catalogue every organism present at each life stage, the model distilled the most representative taxa driving these transitions, providing a biologically meaningful overview of microbial succession. To our knowledge, this is the first time such age‑associated microbial signatures have been described for the chicken nasal niche. By integrating multiple datasets and applying a standardized normalization and feature‑selection workflow, our approach overcomes limitations of previous single‑cohort studies, revealing consistent microbial age signatures at genus‑level resolution across heterogeneous sources. Nevertheless, potential technical variation among sequencing regions or DNA extraction methods should be considered, as these factors could partially influence the detected patterns.

These findings are consistent with previous studies showing that the respiratory microbiota of chickens and turkeys undergoes marked compositional shifts with age, particularly within the upper airways [1, 2]. However, those studies were limited to single-breed cohorts, covered a narrow age range, and lacked the statistical power to disentangle age-related changes from genetic and environmental variability. Importantly, age-dependent microbial dynamics are not confined to the respiratory tract. In the chicken gut, for instance, the cecal microbiome is initially dominated by Enterobacteriaceae and, later transitioning toward Gram-positive Firmicutes (e.g., *Ruminococcus* and *Oscillospira*) by day 14 [39]. Similar successional patterns occur in ducks, where microbial diversity increases and stabilizes during the juvenile period, indicating a general age-related maturation across avian mucosal sites [40], suggesting that similar analytical frameworks could be applied to study microbiota maturation in other anatomical locations, such as the gut microbiota. Consistent with these longitudinal trends, our model pinpointed specific genera whose relative abundances best reflected host age.

Importantly, the predefined age categories used in this study were not arbitrarily defined. They were originally based on reproducible alpha-diversity and compositional shifts described in our previous work [3], including a marked transition between 4 and 5 weeks of age across multiple respiratory sites. A second breakpoint was observed around 50 weeks of age, corresponding to the post-peak (late laying) stage, a biologically meaningful transition well described in laying performance [41, 42] and associated with structural changes in the chicken respiratory microbiota [43].

Our model identified *Corynebacterium*, *Kokuria*, and members of the Micrococcaceae family between other taxa as strong predictors of chicken age. Evidence from previous poultry studies supports their biological relevance. In turkeys, late-emerging dominant taxa in the respiratory tract include *Corynebacterium* and *Deinococcus* [44]. Similarly, as birds mature, the abundance of *Lactobacillus* decreases, while Actinobacteria-dominated genera become increasingly prominent [1]. Collectively, these taxa may represent integral components of a stable, adult-associated respiratory microbiome, potentially contributing to mucosal homeostasis and conferring colonization resistance.

The present study implemented a study-aware, multistep machine-learning framework integrating dimensionality reduction, feature selection, and post hoc interpretability to identify biologically informative taxa. Feature selection was performed within a Leave-One-Study-Out (LOSO) cross-validation scheme using Random Forest–based Recursive Feature Elimination (RFE), ensuring that biomarkers were identified exclusively from training data and were robust to inter-study heterogeneity. By retaining only taxa consistently selected across ≥ 70% of LOSO iterations, we derived a compact and stable consensus signature that preserved classification performance while minimizing overfitting. Model interpretability was further enhanced through Shapley Additive Explanations (SHAP), which quantified both the magnitude and directionality of each taxon’s contribution to age prediction. This integrated RFE–SHAP strategy enabled the reduction of thousands of microbial features to a biologically interpretable core set, providing direct ecological insight into taxa associated with host maturation while maintaining strong predictive accuracy. Moreover, the modular design of the workflow facilitates its extension to alternative classification tasks, including infection status, dietary interventions, or environmental exposures [45]. Despite certain fluctuations in model performance during specific LOSO iterations, these instances were primarily attributed to the high concentration of minority-class samples within those particular cohorts. The exclusion of these data-rich studies during cross-validation likely diminished the training set’s representational quality for those categories. However, by implementing a consensus model based on persistent features, we were able to filter out study-specific noise and isolate biologically relevant signals, thereby significantly enhancing the model’s overall generalizability. To further refine these predictive capabilities, increased access to longitudinal datasets encompassing birds beyond six weeks of age would be essential to fully evaluate the model’s performance during later developmental stages.

Predicting host age from nasal microbiota profiles has both ecological and epidemiological implications. In wild bird populations, age is often unknown at capture; a microbiota-based “clock” could provide non-invasive age estimates, enhancing demographic modeling and pathogen surveillance. For species that serve as reservoirs of avian influenza and other zoonotic agents, integrating microbiota-derived age inference with migration or habitat data could elucidate how host demography shapes pathogen maintenance and transmission. However, applying such microbiota-based clocks to wild systems will require validation across variable dietary, environmental, and ecological contexts to ensure robustness. Such applications would not only broaden the biological relevance of microbiome-based models but also open new avenues for non-invasive wildlife monitoring and conservation-oriented research.

In conclusion, our study presents a robust and interpretable machine learning framework capable of identifying age-dependent microbial signatures within the chicken nasal microbiota across heterogeneous datasets. By integrating normalization, LOSO cross-validation, and SMOTE to address class imbalance, alongside Recursive Feature Elimination (RFE) and SHAP-driven benchmarking, we demonstrated that a compact consensus of taxa can accurately predict host age while maintaining high biological interpretability. Although certain LOSO iterations highlighted the challenges of unbalanced datasets, the use of a consensus-based signature successfully isolated stable biological signals from study-specific noise, enhancing the model’s generalizability. This framework not only advances our understanding of microbiota maturation in poultry but also establishes a transferable analytical pipeline applicable to other species, production systems ecological contexts as well as to other anatomical microbiota such as the gut microbiome. Future research integrating metagenomic and transcriptomic data will help to elucidate the functional mechanisms underlying these microbial transitions and to refine microbiota-based biomarkers for health monitoring, management of poultry production systems, and wildlife surveillance. Together, these findings position interpretable machine learning as a powerful tool for uncovering conserved host-microbe dynamics across avian systems.

## Supplementary Information

Additional file 1: Figure S1. Model calibration and interpretability of the selected classifier. Classification reliability was assessed using a) Windowed Calibration Curves and b) Receiver Operating Characteristic (ROC) curves, evaluating the agreement between predicted probabilities and observed outcomes.

Additional file 2: Table S1. List of publicly available online resources and software packages that served as the methodological basis for each step of the analysis performed in this study.

Additional file 3: Table S2. Microbial taxa identified via RFE across LOSO iterations. Features were retained in the consensus signature only if they persisted in at least 70% of the independent iterations to ensure cross-study stability.

Additional file 4: Table S3. Performance metrics for machine learning architectures evaluated on the Consensus Core. Results were obtained via 10-fold stratified cross-validation and are presented as the mean ± standard error.

Additional file 5. Trained Random Forest model for chicken age classification. The model is provided in .rds format.

Additional file 6. R script to run Random Forest trained model predictions.

## Data Availability

The datasets analysed during the current study are available in the NCBI BioProject repository, under the following accession numbers PRJNA393945, PRJNA597337, PRJNA668323, PRJNA796490 and PRJNA509940. The validation dataset is available in (https://doi.org/10.5281/zenodo.17466208). The trained Random Forest model and the script to run it are provided as additional files.

## Abbreviations

ASV: Amplicon Sequence Variant
AUC: Area Under the Curve
BSL-3: Biosafety Level 3
CART: Classification and Regression Trees
CPM: Counts Per Million
DPI: Days Post-Infection
HPAIV: Highly Pathogenic Avian Influenza Virus
kNN: K-Nearest Neighbors
LPAI: Low-Pathogenic Avian Influenza
LOSO: Leave-One-Study-Out
ML: Machine Learning
MASHV: Mean Absolute SHAP Value
PCA: Principal Component Analysis
RFE: Recursive Feature Elimination
RF: Random Forest
RMSE: Root Mean Square Error
SHAP: SHapley Additive exPlanations
SMOTE: Synthetic Minority Over-sampling Technique
SPF: Specific Pathogen-Free
SVM: Support Vector Machine
XGBoost: Extreme Gradient Boosting
