# Supplementary figures and images for: Respiratory microbiota maturation enables machine learning based age prediction in chickens
Source: BMC Microbiol. 2026 Mar 18;26:400. doi: 10.1186/s12866-026-04947-3 (PMC13112838; doi:10.1186/s12866-026-04947-3)

**a**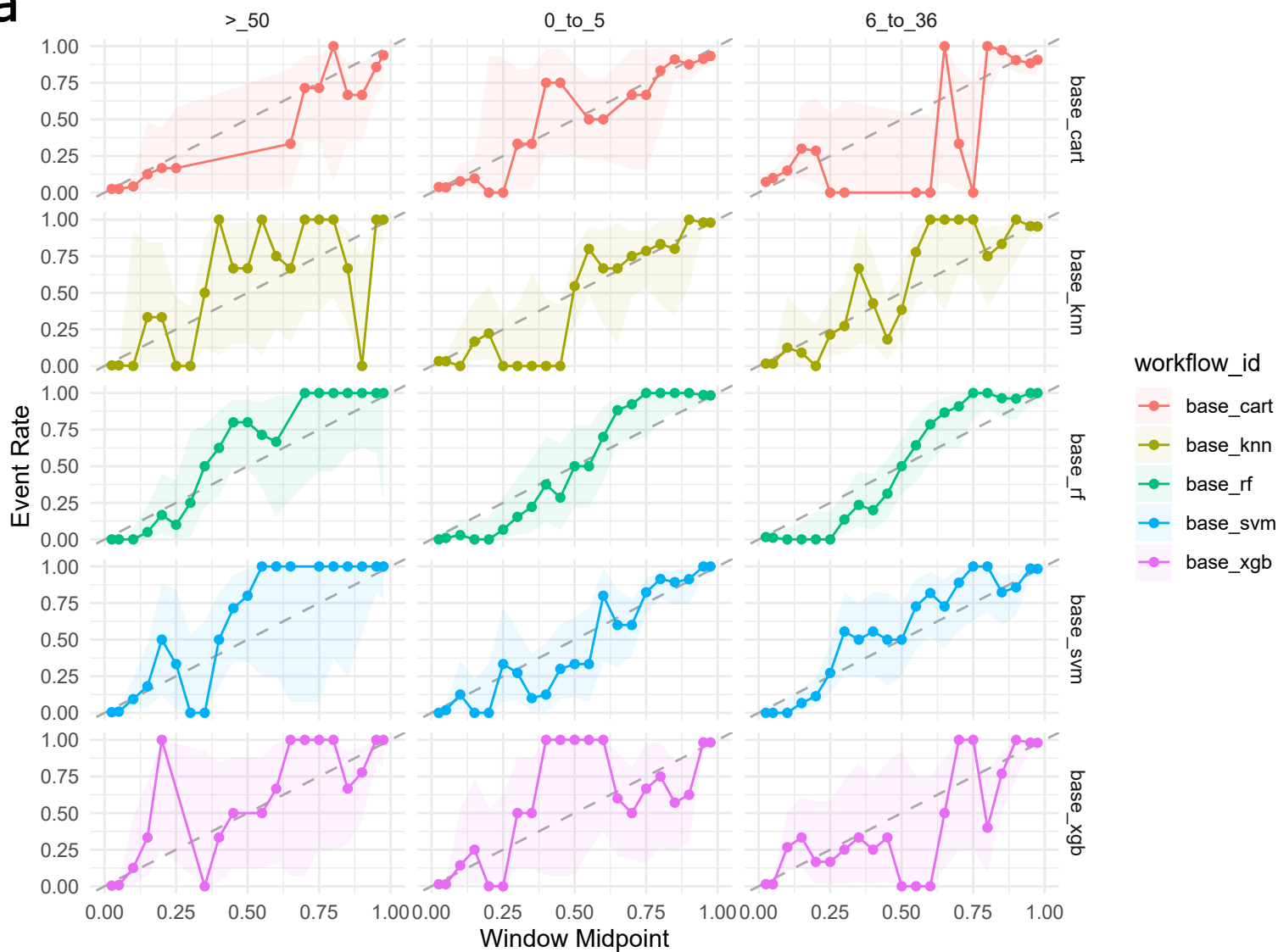**b**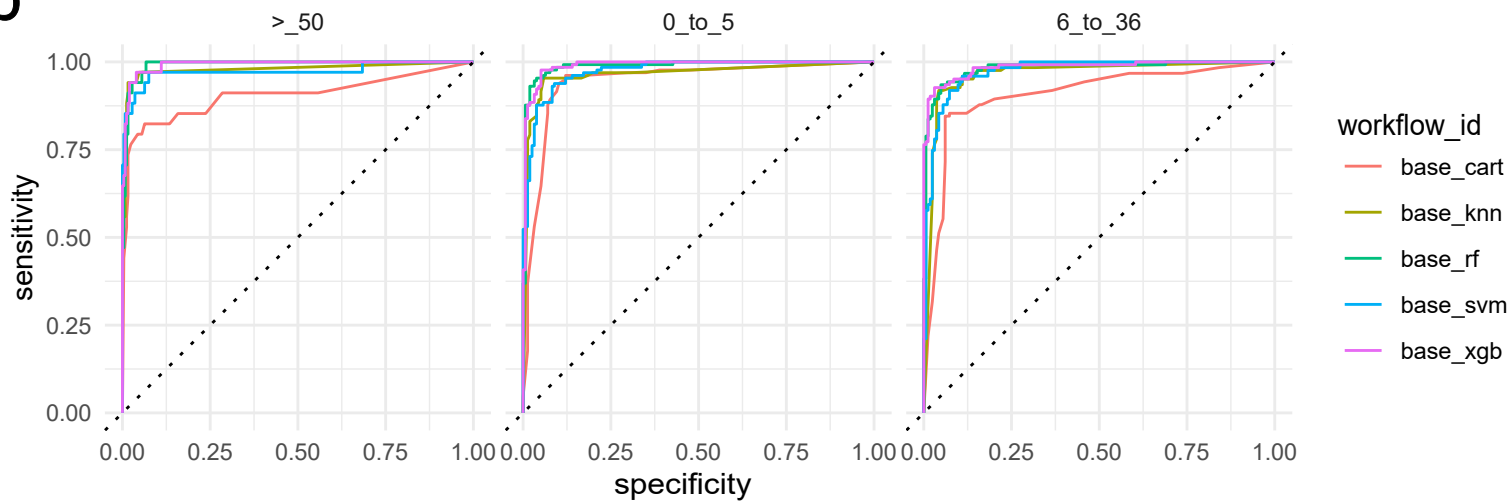

Supplement: Supplementary file 1 — Additional file 1: Figure S1. Model calibration and interpretability of the selected classifier. Classification reliability was assessed using a) Windowed Calibration Curves and b) Receiver Operating Characteristic (ROC) curves, evaluating the agreement between predicted probabilities and observed outcomes. [file 12866_2026_4947_MOESM1_ESM.pdf]
